# Improving survival of 3760 patients with lymphoma: Experience of an academic center over two decades

**DOI:** 10.1002/cam4.3037

**Published:** 2020-04-12

**Authors:** Weiping Liu, Xinqiang Ji, Yuqin Song, Xiaopei Wang, Wen Zheng, Ningjing Lin, Meifeng Tu, Yan Xie, Lingyan Ping, Zhitao Ying, Chen Zhang, Lijuan Deng, Meng Wu, Feier Feng, Xin Leng, Yingli Sun, Tingting Du, Jun Zhu

**Affiliations:** ^1^ Key Laboratory of Carcinogenesis and Translational Research (Ministry of Education) Department of Lymphoma Peking University Cancer Hospital & Institute Haidian District Beijing China; ^2^ Key Laboratory of Carcinogenesis and Translational Research (Ministry of Education) Department of Medical Record Statistics Peking University Cancer Hospital & Institute Haidian District Beijing China

**Keywords:** Hodgkin disease, lymphoma, lymphoma, non‐Hodgkin, prognosis, survival

## Abstract

**Background:**

The treatment outcomes and prognosis of lymphoma are affected by various factors such as hospital types. This study was to describe the temporal trend in the survival of lymphoma in an academic center in China.

**Methods:**

A total of 3840 consecutive patients with lymphoma diagnosed between 1996 and 2015 were reviewed. Eighty patients were excluded, and finally, 3760 patients were analyzed in this study. The cohort was divided into four groups according to calendar periods at diagnosis: 1996‐2000, 2001‐2005, 2006‐2010, and 2010‐2015. The overall survival (OS) rates among the four groups were compared.

**Results:**

The 5‐ and 10‐year OS for the whole cohort were 62% and 52%, respectively. The 5‐year OS of patient with classic Hodgkin lymphoma (cHL), mature B‐cell lymphoma (BCL), and peripheral T‐cell lymphoma (PTCL) were 79%, 63%, and 50%, respectively. Among mature BCL, the 5‐year OS was highest in follicular lymphoma (77.8%), followed by Burkitt lymphoma (76.5%), marginal zone lymphoma (74.1%), diffuse large B‐cell lymphoma (61.5%), small lymphocytic lymphoma/chronic lymphocytic leukemia (55.1%), and mantle cell lymphoma (44.3%). Among PTCL, the 5‐year OS was highest in ALK+anaplastic large cell lymphoma (79.0%), followed by ALK−anaplastic large cell lymphoma (63.1%), natural killer/T‐cell lymphoma (57.7%), angioimmunoblastic T‐cell lymphoma (34.9%, and peripheral T‐cell lymphoma not otherwise specified (27.6%). Significant improvement in the survival of lymphoma was observed, with the 5‐year OS increasing from 48% in 1996‐2000 to 65% in 2011‐2015 (*P* < .001). The 5‐year OS of patients with cHL, mature BCL, and PTCL changed from 55%, 49%, and 41% in 1996‐2000 to 79%, 65%, and 51% in 2011‐2015, respectively (*P* values were .014, .002, and .592, respectively).

**Conclusion:**

The survival of most types of lymphoma such as cHL and mature BCL, rather than PTCL, was improved significantly during the past two decades.

## Graphical abstract

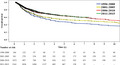

## 1 INTRODUCTION

Lymphoma is classified roughly into Hodgkin lymphoma (HL) and non–Hodgkin lymphoma (NHL), according to the World Health Organization classification of lymphoid neoplasms.^1^ Lymphoma is a common leading cause of death worldwide. According to the systematic analysis for the Global Burden of Disease Study 2016,^2^ an estimated 28 700 deaths globally was due to HL and 239 600 was due to NHL, respectively. It was estimated 52 000 deaths due to lymphoma and myeloma in China in 2017, with an increase by 4.5% annually during the period 2004‐2016.^3^ The worst 5‐year relative survival was observed in peripheral T‐cell lymphomas (PTCL), which varied from 36% to 56%.^4^

Based on data from the National Central Cancer Registry of China, the age‐standardized 5‐year relative survival of lymphoid neoplasms increased from 32.6% during 2003‐2005 to 37.2% during 2012‐2015.^5^ Because the hospital type was an important factor influencing treatment outcomes, differentiated care quality and prognosis could be found in different cancer centers. For example, a multicenter study^6^ involving 1332 patients with nature killer/T‐cell lymphoma demonstrated that the 5‐year overall survival (OS) varied from 58% to 70%, according to the dose of radiotherapy. Moreover, the data regarding comprehensive survival description of lymphoma with temporal trend are rare in China. In this real‐world study, we sought to describe the survival of lymphoma in our academic center, and evaluate the temporal trend over two decades.

## 2 PATIENTS AND METHODS

This retrospective study was approved by the Ethics Committee at Peking University Cancer Hospital and Institute, in accordance with the Declaration of Helsinki; and the requirement for informed consent was waived because of the anonymous nature of the data.

A total of 3840 patients with de novo lymphoma diagnosed from January 1, 1996 to December 31, 2015 were retrospectively collected. Eighty patients were excluded from the study for the following reasons: unclassified pathological type (n = 24), incomplete clinical data (n = 16), and only best supportive care (n = 40). Finally, 3760 patients were analyzed in this study. The clinical and histological data of these patients were collected, including age, gender, pathology type, Ann‐Arbor stage, and follow‐up information. All patients in the study were followed up until death or the last visit at our institute, with a date of final data collection of June 30, 2018. Death was confirmed by medical record review or telephone survey. OS was calculated from the time of diagnosis to death or the end of the follow‐up period. All‐cause death time was defined as the time from diagnosis to death as a result of any cause. The cohort was divided into four subgroups according to calendar periods at diagnosis: 1996‐2000, 2001‐2005, 2006‐2010, and 2010‐2015. Causes of death were classified as lymphoma‐specific diseases, cardiovascular diseases, secondary malignancies, and other causes.

All statistical analyses were performed using the IBM SPSS Statistics for Windows (Version 21.0; IBM Corp., New York, USA) and R version 3.5.3 (Institute for Statistics and Mathematics, Vienna, Austria; www.R‐project.org) with R packages, cmprsk, and survminer. Pearson chi‐square analysis or Fisher's exact test was used to compare the difference of categorical variables. The *t* test or Kruskal‐Wallis test was used to compare the difference of continuous variables. The differences in OS among the groups were compared by the Kaplan‐Meier curves with log‐rank chi‐square test. The cumulative probability of all‐cause death and competing causes of death were calculated using nonparametric cumulative incidence functions (CIF). All statistical tests were two‐tailed, and *P* < .05 was considered statistically significant.

## 3 RESULTS

### 3.1 Baseline characteristics

Table 1 summarizes the characteristics of lymphoma patients during different periods. Totally, male predominance was observed with a male‐to‐female ratio of 1.4:1. The median age of the whole cohort was 52.3 (range, 7.3‐92.7) years, and there was an increase in the median age from 1996‐2000 to 2011‐2015 (*P* = .038). Of the whole cohort, 87.0% had NHL, and 13.0% had HL. A higher proportion of NHL was observed in 2001‐2005. There were 61.8% of patients at advanced stage (stage 3‐4) and 38.2% at early stage (stage 1‐2). An increase in proportions of early‐stage patients was observed from 1996‐2000 to 2011‐2015.

**Table 1:** Baseline characteristics of 3760 patients with lymphoma

Characteristics	Number (%)				*P* value
	1996 − 2000 (N = 166)	2001 − 2005 (N = 361)	2006 − 2010 (N = 1072)	2011 − 2015 (N = 2161)	
Sex					.230
Male	108 (65.1)	217 (60.1)	619 (57.7)	1242 (57.5)	
Female	58 (34.9)	144 (39.9)	453 (42.3)	919 (42.5)	
Age (y)					.038
Median (range)	45.8 (12.8−84.3)	50.0 (12.2−88.3)	48.4 (7.3−90.8)	51.1 (11.1−92.7)	
Histology					.019
HL	25 (15.1)	29 (8.0)	135 (12.6)	299 (13.8)	
NHL	141 (84.9)	332 (92.0)	937 (87.4)	1862 (86.2)	
LBL	1 (0.6)	7 (1.9)	62 (5.8)	79 (3.7)	
Mature BCL	85 (51.2)	225 (62.3)	688 (64.2)	1446 (66.9)	
FL	8 (4.8)	8 (2.2)	54 (5.0)	180 (8.3)	
SLL	3 (1.8)	3 (0.8)	23 (2.2)	33 (1.5)	
MZL	0 (0)	4 (1.1)	46 (4.1)	141 (6.5)	
BL	1 (0.6)	0 (0)	9 (0.8)	20 (0.9)	
DLBCL	38 (22.9)	174 (48.2)	501 (46.7)	920 (42.6)	
MCL	0 (0)	3 (0.8)	38 (3.6)	85 (3.9)	
Others	35 (21.1)	33 (9.1)	17 (1.6)	67 (3.1)	
PTCL	55 (33.1)	100 (27.7)	187 (17.4)	337 (15.6)	
ALK+ALCL	1 (0.6)	7 (1.9)	14 (1.3)	38 (1.8)	
ALK−ALCL	0 (0)	1 (0.2)	6 (0.6)	27 (1.2)	
NKTCL	1 (0.6)	40 (11.1)	93 (8.7)	154 (7.1)	
AITL	3 (1.8)	5 (1.4)	29 (2.7)	57 (2.6)	
PTCL NOS	2 (1.2)	12 (3.3)	25 (2.3)	30 (1.4)	
Others	47 (28.3)	29 (8.0)	12 (1.1)	31 (1.4)	
Stage					<.001
1‐2	44 (26.5)	122 (33.8)	390 (36.4)	879 (40.7)	
3‐4	122 (73.5)	239 (66.2)	682 (63.6)	1282 (59.3)	

AITL, angioimmunoblastic T‐cell lymphoma; ALK+ALCL, ALK+ anaplastic large cell lymphoma; ALK−ALCL, ALK− anaplastic large cell lymphoma; BCL, B‐cell lymphoma; BL, Burkitt lymphoma; DLBCL, diffuse large B‐cell lymphoma; FL, follicular lymphoma; HL, Hodgkin lymphoma; LBL: lymphoblastic lymphoma; MCL, mantle cell lymphoma; MZL, marginal zone lymphoma; NHL, non–Hodgkin lymphoma; NKTCL, natural killer/T‐cell lymphoma; PTCL: peripheral T‐cell lymphomas; PTCL NOS, peripheral T‐cell lymphoma not otherwise specified; SLL, small lymphocytic lymphoma/chronic lymphocytic leukemia.

### 3.2 Overall survival

**Figure 1:**
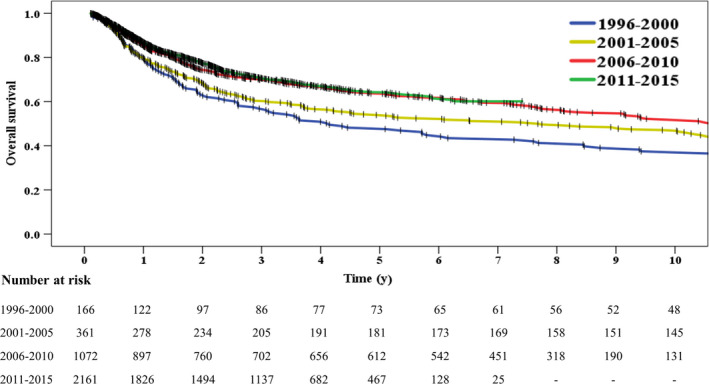
Increase in overall survival of lymphoma from 1996 to 2015

For the whole cohort, the 5‐ and 10‐year OS were 62% and 52%, respectively. The duration of follow‐up among four groups were different, with the median follow‐up of 10.5 years in the 1996 − 2000 group, 10.3 years in the 2001‐2005 group, 8.1 years in the 2006‐2010 group, and 3.8 years in the 2011‐2015 group. Figure 1 illustrates the Kaplan‐Meier curves of the four groups, with statistically significant increases in OS over time (*P* < .001). The 5‐year OS increased from 48% in the 1996‐2000 group to 65% in the 2011‐2015 group. The 10‐year survival rate was not available in the 2011‐2015 group, but the difference was significant among 1996‐2000, 2001‐2005, and 2006‐2010 groups (38% vs 47% vs 52%, *P* < .001).

There were 488 cases of HL, of which 11 were nodular lymphocyte‐predominant HL (NLPHL) and 477 were classic HL (cHL). The 5‐ and 10‐year OS were 80% and 71% in the whole cohort, 100% and 67% in the NLPHL group, and 79% and 71% in the cHL group, respectively. In the cHL group, the 5‐ and 10‐year OS were 89% and 86% for those at early stage, and 72% and 62% for those at advanced stage, respectively (*P* < .001). The 5‐year OS of patients with cHL increased from 55.4% in the 1996‐2000 group to 79.0% in the 2010‐2015 group (*P* = .014, Table 3).

There were 3272 cases of NHL, of which 149 (4.6%) were lymphoblastic lymphoma (LBL), 2444 (74.7%) were mature B‐cell lymphoma (BCL), and 679 (20.7%) were PTCL. The 5‐ and 10‐year OS were 60% and 49% in the whole cohort, 48% and 42% in the LBL group, 63% and 52% in the mature BCL group, and 50% and 41% in the PTCL group, respectively.

**Figure 2:**
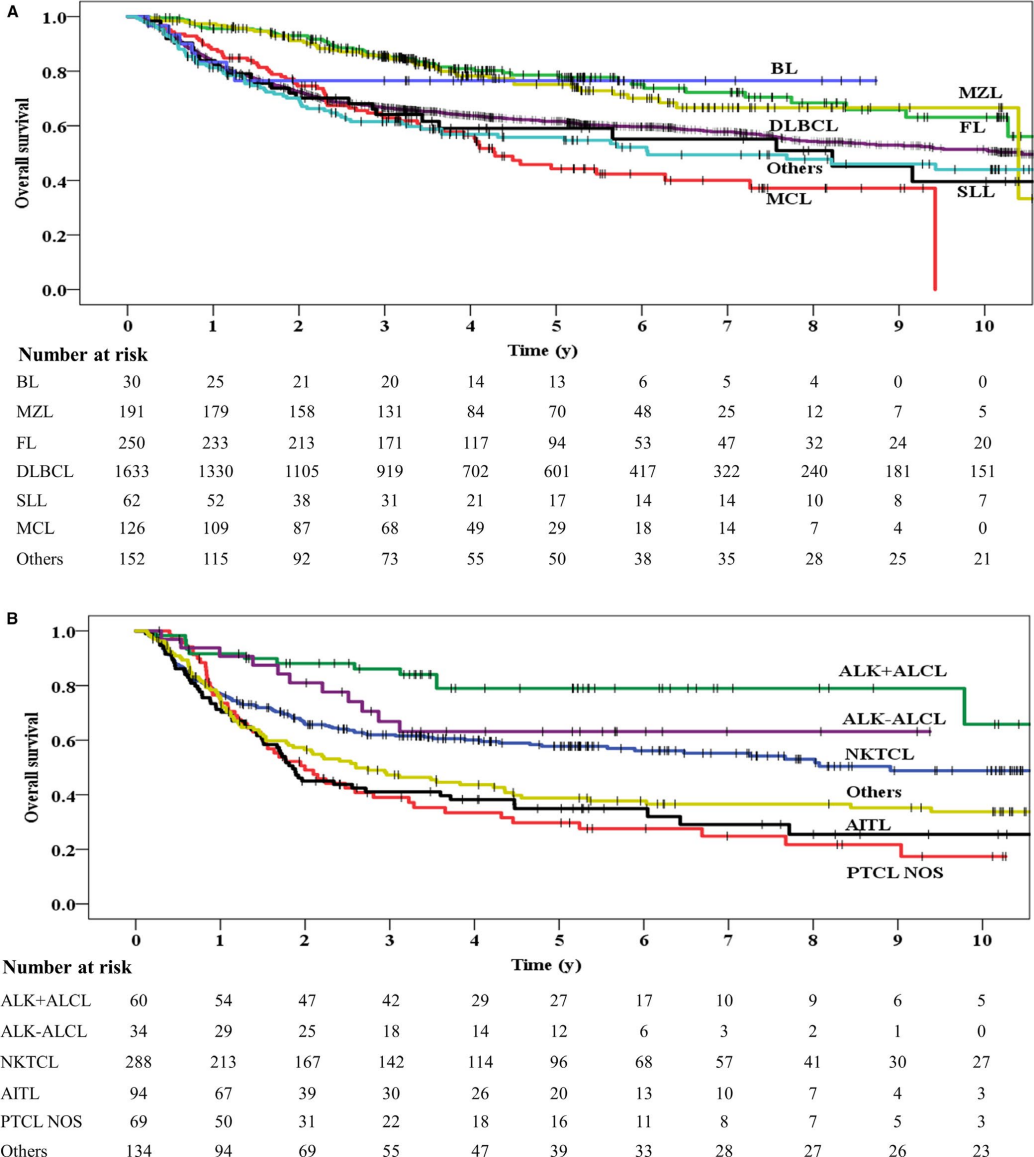
Overall survival for mature B‐cell lymphoma (A) and peripheral T‐cell lymphoma (B). AITL, angioimmunoblastic T‐cell lymphoma; ALK+ALCL, ALK+ anaplastic large cell lymphoma; ALK−ALCL, ALK− anaplastic large cell lymphoma; BL, Burkitt lymphoma; DLBCL, diffuse large B‐cell lymphoma; FL, follicular lymphoma; SLL, small lymphocytic lymphoma/ chronic lymphocytic leukemia; MCL, mantle cell lymphoma; MZL, marginal zone lymphoma; NKTCL, natural killer/T‐cell lymphoma; PTCL NOS, peripheral T‐cell lymphoma not otherwise specified

For mature BCL, there was an increase in 5‐year OS of more than 10 percentage point from 1996‐2000 to 2010‐2015 (Table 2). Figure 2A shows the difference in survival outcomes in the mature BCL group. Totally, those patients with indolent B‐cell lymphomas had better 5‐year OS than those with aggressive B‐cell lymphomas (55% vs 48%, *P* < .001). The 5‐year OS was highest in follicular lymphoma (FL, 77.8%), followed by Burkitt lymphoma (76.5%), marginal zone lymphoma (74.1%), DLBCL (61.5%), small lymphocytic lymphoma/chronic lymphocytic leukemia (55.1%), and mantle cell lymphoma (44.3%). Notably, the 5‐year OS of patients with diffuse large B‐cell lymphoma (DLBCL) in the four different periods were 47.2%, 53.1%, 62.6%, and 63.4%, respectively (*P* = .031). Compared with the DLBCL patients received chemotherapy without anthracyclines (n = 112), those DLBCL patients received chemotherapy with anthracyclines (n = 1521) had better survival outcome (5‐year OS, 63.0% vs 41.2%; 10‐year OS, 52.6% vs 25.4%; *P* < .001). Among the 1521 DLBCL patients received chemotherapy with anthracyclines, those treated with rituximab had better survival outcome than those treated without rituximab (5‐year OS, 69.0% vs 51.7%; 10‐year OS, 55.6% vs 43.9%; *P* < .001).

**Table 2:** Comparison of 5‐year overall survival of patients with cHL, BCL and PTCL among 4 groups

Category	5‐year overall survival (%)				*P* value
	1996‐2000	2001‐2005	2006‐2010	2011‐2015	
cHL (N = 477)	55.4	71.4	84.9	79.0	.014
Mature BCL (N = 2444)	48.9	54.6	63.6	65.3	.002
FL (N = 250)	42.9	87.5	77.4	77.6	.518
SLL (N = 62)	33.3	33.3	53.5	64.8	.826
MZL (N = 191)	−	50.0	79.0	72.0	.508
BL (N = 30)	−	−	77.8	74.7	.864
DLBCL (N = 1633)	47.2	53.1	62.6	63.4	.031
MCL (N = 126)	−	33.3	37.5	52.5	.931
Others (N = 152)	46.0	56.4	56.7	60.9	.656
PTCL (N = 679)	40.8	46.3	52.6	50.6	.592
ALK+ALCL (N = 60)	−	57.1	84.6	76.7	.750
ALK−ALCL (N = 34)	−	0	60	66.4	.481
NKTCL (N = 288)	−	61.6	59.7	53.0	.582
AITL (N = 94)	0	20.0	40.8	36.5	.021
PTCL NOS (N = 69)	50.0	25.0	21.8	33.8	.579
Others (N = 134)	37.0	30.1	41.7	37.5	.677

AITL, angioimmunoblastic T‐cell lymphoma; ALK+ALCL, ALK+ anaplastic large cell lymphoma; ALK−ALCL, ALK− anaplastic large cell lymphoma; BCL, B‐cell lymphoma; BL, Burkitt lymphoma; cHL, classic Hodgkin lymphoma; DLBCL, diffuse large B‐cell lymphoma; FL, follicular lymphoma; LBL: lymphoblastic lymphoma; MCL, mantle cell lymphoma; MZL, marginal zone lymphoma; NKTCL, natural killer/T‐cell lymphoma; PTCL: peripheral T‐cell lymphomas; PTCL NOS, peripheral T‐cell lymphoma not otherwise specified; SLL, small lymphocytic lymphoma/chronic lymphocytic leukemia

**Table 3:** Cause of death

	Number (%)				
	Primary disease	Secondary malignancy	Cardiovascular disease	Others	Unknown
Total (N = 1424)	1147 (80.5)	47 (3.3)	61 (4.3)	108 (7.6)	61 (4.3)
*Time*					
1996‐2000	101 (99.0)	0 (0)	0 (0)	0 (0)	1 (1.0)
2001‐2005	184 (92.0)	2 (1.0)	4 (2.0)	4 (2.0)	6 (3.0)
2006‐2010	358 (79.7)	15 (3.3)	23 (5.1)	37 (8.3)	16 (3.6)
2011‐2015	504 (74.9)	30 (4.5)	34 (5.1)	67 (9.9)	38 (5.6)
*Histology*					
HL (N = 165)	134 (9.4)	5 (0.4)	6 (0.4)	16 (1.1)	4 (0.3)
NHL (N = 1259)	1013 (71.7)	42 (2.9)	55 (3.9)	92 (6.5)	57 (4.0)
LBL (N = 60)	48 (3.4)	1 (0.1)	4 (0.3)	4 (0.3)	3 (0.2)
Mature BCL (N = 933)	744 (52.3)	34 (2.4)	40 (2.8)	71 (5.0)	44 (3.1)
FL (N = 94)	76 (5.3)	3 (0.2)	5 (0.4)	5 (0.4)	94 (6.6)
SLL (N = 25)	18 (1.3)	2 (0.1)	2 (0.1)	2 (0.1)	1 (0.1)
MZL (N = 62)	52 (3.7)	2 (0.1)	3 (0.2)	2 (0.1)	3 (0.2)
BL (N = 10)	6 (0.4)	0 (0)	2 (0.1)	1 (0.1)	1 (0.1)
DLBCL (N = 618)	487 (34.2)	25 (1.8)	24 (1.7)	52 (3.7)	30 (2.1)
MCL (N = 39)	28 (2.0)	1 (0.4)	4 (0.3)	4 (0.3)	2 (0.1)
Others (N = 85)	77 (5.4)	1 (0.1)	0 (0)	5 (0.4)	2 (0.1)
PTCL (N = 266)	221 (15.5)	7 (0.5)	11 (0.8)	17 (1.2)	10 (0.7)
ALK+ALCL (N = 16)	15 (1.1)	0 (0)	0 (0)	1 (0.1)	0 (0)
ALK−ALCL (N = 11)	6 (0.4)	1 (0.1)	1 (0.1)	3 (0.2)	0 (0)
NKTCL (N = 110)	85 (6.0)	3 (0.2)	10 (0.7)	8 (0.6)	4 (0.3)
AITL (N = 30)	24 (1.7)	1 (0.1)	0 (0)	2 (0.1)	3 (0.2)
PTCL NOS (N = 26)	24 (1.7)	1 (0.1)	0 (0)	1 (0.1)	0 (0)
Others (N = 73)	67 (4.7)	1 (0.1)	0 (0)	2 (0.1)	3 (0.2)

AITL, angioimmunoblastic T‐cell lymphoma; ALK+ALCL, ALK+ anaplastic large cell lymphoma; ALK−ALCL, ALK− anaplastic large cell lymphoma; BCL, B‐cell lymphoma; BL, Burkitt lymphoma; DLBCL, diffuse large B‐cell lymphoma; FL, follicular lymphoma; HL, Hodgkin lymphoma; LBL: lymphoblastic lymphoma; MCL, mantle cell lymphoma; MZL, marginal zone lymphoma; NHL, non–Hodgkin lymphoma; NKTCL, natural killer/T‐cell lymphoma; PTCL: peripheral T‐cell lymphomas; PTCL NOS, peripheral T‐cell lymphoma not otherwise specified; SLL, small lymphocytic lymphoma/chronic lymphocytic leukemia

During the study period of 1996‐2015, the 5‐year OS of patients with PTCL ranged from 40.8% to 52.6% without statistical significance (Table 2). Figure 2B shows the difference in survival outcomes in the PTCL group. The 5‐year OS was highest in anaplastic lymphoma kinase‐positive (ALK+) anaplastic large cell lymphoma (79.0%), followed by ALK−anaplastic large cell lymphoma (63.1%), natural killer/T‐cell lymphoma (57.7%), angioimmunoblastic T‐cell lymphoma (34.9%), and peripheral T‐cell lymphoma not otherwise specified (27.6%).

### 3.3 Causes of death

During the follow‐up period, 1424 patients died, including 165 cases of HL and 1259 cases of NHL (Table 3). Primary disease (80.5%) was the most common cause of death, followed by cardiovascular diseases (4.3%) and secondary malignancies (3.3%). Lymphoma‐specific diseases, cardiovascular diseases and secondary malignancies accounted for 82.8%, 3.3%, and 2.7% of causes of death in those patients who survived less than 5 years, and 60.8%, 12.8%, and 8.1% of causes of death in those surviving more than 5 years, respectively. Moreover, the proportion of lymphoma‐specific death decreased from 99.0% in the 1996‐2000 group to 74.9% in the 2011‐2015 group, while the proportion of cardiovascular disease death and secondary malignancy death increased from 0% to 9.6% (Table 3).

**Figure 3:**
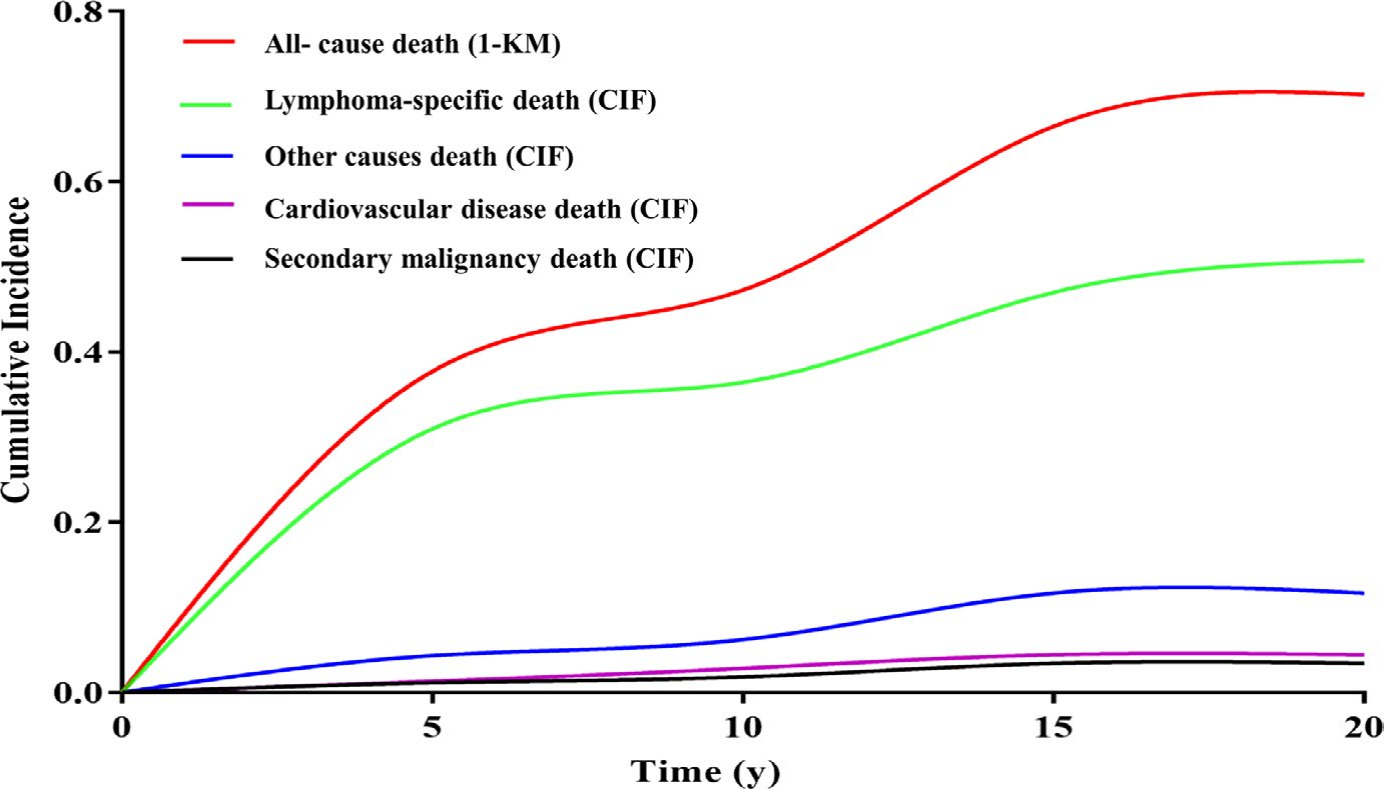
Cumulative incidences of competing causes of death in patients with lymphoma. KM, Kaplan‐Meier; CIF, cumulative incidence function

The 5‐year and 10‐year CIF were 37.7% (95% confidence interval [CI], 37.0% to 38.4%) and 47.3% (95% CI, 47.0% to 47.6%) for all‐cause death, 30.9% (95% CI, 30.6% to 31.2%) and 36.4% (95% CI, 36.1% to 36.7%) for lymphoma‐specific death, 1.3% (95% CI, 1.0% to 1.6%) and 2.8% (95% CI, 2.4% to 3.2%) for cardiovascular disease death, 1.1% (95% CI, 0.9% to 1.3%) and 1.8% (95% CI, 1.6% to 2.0%) for secondary malignancy death, and 4.3% (95% CI, 4.0% to 4.6%) and 6.2% (95% CI, 5.2% to 7.2%) for other causes of death, respectively (Figure 3).

## 4 DISCUSSION

Based on data of the global surveillance of trends in cancer survival 2000−2014 from CONCORD‐3 study, the 5‐year relative survival rate of lymphoid neoplasms in China was lower remarkably than that in European countries (38.3% vs 70% or higher) during the same period of 2000‐2014.^7^ However, treatment outcomes and survival of lymphoma were affected by socioeconomic status and hospital type. In the present study, a notably improved outcome for patients with lymphoma was observed in our academic center over the past 20 years, with the 5‐year OS of 62% for the whole cohort, which seemed to be much better than that nationally. Many factors could contribute to the dramatic improvements. First, the access to healthcare system was improved over the past two decades. For example, the number of hospital beds and the number of doctors per 1000 people were 2.53 and 1.56 in 1990, increasing to 3.31 and 1.75 in 2009.^8^ Second, the China government adjusted health insurance systems with a coverage over 95.7% on population of 1.37 billion until 2011.^9^ Third, the treatment of lymphoma in those tertiary hospitals like our academic center preferably complied with clinical practice guidelines and risk‐adapted individualizing strategy.^10^ These findings strongly highlighted the need of standardized procedures of diagnosis and treatment, particularly for those nonacademic centers in China.

The survival of HL has improved dramatically over the past 50 years, which was mainly due to the development of chemotherapy regimens and advancement in radiotherapy techniques.^11^ Conventional combination of chemotherapy and radiotherapy could cure the majority of patients with newly diagnosed HL, while salvage therapy such as hematopoietic stem‐cell transplantation^12^ gave the second chance of cure for patients with relapsed or refractory disease. In a recent study,^13^ the 5‐year OS of patients with primary nodal Hodgkin lymphoma and primary extranodal classical were 97.7% and 64.6% during the study period of 2008‐2018. A study^14^ from the British National Lymphoma Investigation involving 6834 HL patients demonstrated that the 5‐ and 10‐year OS increased from 73.5% and 62.4% in the 1970s to 93.8% and 89.6% in the 2000s, with a reduced risk of second malignancy and cardiac‐related deaths over time. A study^15^ based on the database of Surveillance, Epidemiology, and End Results (SEER) analyzed 43 330 cHL patients between 1983 and 2014 and found that the 5‐ and 10‐year OS rates were 79.8% and 72.3%, respectively. Similarly, the 5‐year OS of patients with cHL increased by 24% during the past two decades in the present study, which confirmed the benefits for HL from advances in modern treatment techniques.

Immunochemotherapy containing rituximab and anthracyclines not only led to improvement of mature BCL prognosis,^16^, ^17^ but also was proven to be cost‐effective.^18^, ^19^ A study^20^ from SEER database indicated that there were 279 704 cumulative life years saved after introducing rituximab into clinical practice, with 200 278 for DLBCL, 68 177 for FL, and 12 363 for chronic lymphocytic leukemia. In the present study, mature BCL had the best survival among all subtypes of NHL, with more than 10 percentage point increase in 5‐year OS during the follow‐up period, which was partly due to the introduction of rituximab‐based on the recommendation of clinical practice guidelines.^21^ Especially for DLBCL, the proportion of patients receiving rituximab‐containing therapy increased significantly since the Food and Drug Administration of China approved rituximab for the treatment of DLBCL and FL in 2000. Accordingly, the survival of DLBCL was improved with the introduction of rituximab, and the best survival was observed in those patients who received rituximab‐anthracyclines‐based chemotherapy with an increase of 17.3% in 5‐year OS. Although the benefit of immunochemotherapy for mature BCL such as DLBCL has been proven in many prospective trials from Western countries, we confirmed the benefit in a large scale of China patient population by a real world study. Further studies focusing on critical factors such as cost‐effectiveness and long‐term toxicity should be executed.

Although anthracyclines remained an important component of initial therapy for PTCL, therapeutic clinical trials were recommended as initial treatment in clinical practice guidelines,^22^, ^23^ because there was no clear standard of care until now. Except for ALK+anaplastic large cell lymphoma, most of PTCL exhibited poor long‐term prognosis. A multicenter cohort study^24^ which enrolled 499 patients with aggressive PTCL showed that the estimated median survival was 43.0 months, with the 2‐year OS of 58.7%. A report^25^ from the International T‐Cell Project involving 937 patients with PTCL demonstrated that 47% were identified as refractory and 21% as relapsed after first‐line therapy. For those refractory and relapsed patients, the 3‐year OS were 21% and 28%, respectively. A retrospective study^26^ indicated that the estimated 3‐year OS was 43.6% for 116 patients with treatment‐naive PTCL, and the introduction of etoposide in the anthracycline‐based chemotherapy brought benefit to survival. In addition, those patients with PTCL achieving complete remission after first‐line therapy may benefit from autologous hematopoietic stem cell transplantation.^27^ Unfortunately, the 5‐year OS of patients with PTCL was not improved during the past two decades in the present study. These data suggested that more efficient treatment protocols with better survival benefit should be explored for patients with PTCL, and more novel targeted agents should be developed in the future.

Another concern was cause of death due to other diseases such as secondary malignancies. A study^28^ based on SEER‐Medicare Data indicated that the 5‐year other‐cause mortality was 16.2% for aggressive NHL and 17.9% for indolent NHL, respectively, and increased with age and comorbidity level for most subtypes. A multi‐institutional retrospective cohort study^29^ demonstrated that 35% of observed deaths among 2742 survivors of HL were due to secondary malignant neoplasms, and 14% were due to cerebrovascular and heart diseases. In the present study, the proportion of death due to primary disease decreased sharply by 25% during the past two decades, mainly because of the improving antitumor models and supportive treatment. However, primary disease still constructed the majority of causes of death and led to competing risk of other causes of death, which may be one important reason for lower proportion of other causes of death such as cardiovascular diseases and secondary malignancies in the present study. The relatively short follow‐up time in the present study may also contribute to the lower proportion of other causes of death. Notably, the proportion of cardiovascular diseases and secondary malignancies increased by about 10% during the past two decades, and it was higher significantly in those patients who survived more than 5 years. Therefore, given the relatively long‐term survival potential of lymphoma, less toxic therapies should have priority to be taken into consideration when treatment strategy was launched.

Our study is bound by certain limitations typical for a single‐center retrospective analysis. First, we have very quickly become a referral center focusing on diagnosis and treatment of lymphoma since 2006, which resulted in a large number of clinics and admissions annually. Therefore, our results cannot be directly transferred to other academic or nonacademic centers. Second, histology confirmation was not possibly executed due to both the large number of pathological samples and constant changing criteria of pathological classification during two decades. Third, a higher proportion of patients at early stage were diagnosed over time due to advances in examination technology such as positron emission tomography, which possibly masked or diminished significant trends. Finally, the classification of lymphoid neoplasms was revised in 1994, 2001, 2008, and 2016, respectively, and the changes of revision should also be considered in a cautious interpretation of the temporal trends.

## 5 CONCLUSIONS

This study was a collection of data from an academic center over the past two decades and enriched the data currently available about long‐term survival outcomes of lymphoma in China. The stratification of data into four groups over time allowed comparison of temporal trend in survival. The survival was improved significantly from 1996‐2000 to 2011‐2015, especially for HL and BCL, rather than PTCL. These results highlight the need to focus on improvement of survival, especially in patients with PTCL. In addition, secondary malignancies and cardiovascular diseases play an important role in death pattern, which allows insight into the balance between disease control and long‐term toxicity in the treatment decision‐making process for patients with lymphoma.

## 6 ETHICS STATEMENT

The Institutional Review Board of Peking University Cancer Hospital & Institute approved this retrospective study and waived the need for patient informed consent.

## CONFLICT OF INTEREST

The authors declare no potential conflicts of interest.

## AUTHORS’ CONTRIBUTIONS

WL, XJ, and JZ conceived the study and wrote the manuscript; YS, XW, WZ, NL, MT, YX, LP, ZY, CZ, LD, MW, FF, XL, YS, and TD collected data and critically revised the manuscript; WL and XJ performed statistical analyses. All authors read and approved the final manuscript.

## Data Availability

All datasets generated for this study are included in the manuscript.

## References

[1] Swerdlow SH , Campo E , Pileri SA , et al. The 2016 revision of the World Health Organization classification of lymphoid neoplasms. Blood. 2016;127:2375‐2390. 10.1182/blood-2016-01-643569. 26980727 PMC4874220

[2] GBD 2016 Causes of Death Collaborators . Global, regional, and national age‐sex specific mortality for 264 causes of death, 1980–2016: a systematic analysis for the Global Burden of Disease Study 2016. Lancet 2017;390:1151‐1210. doi: 10.1016/S0140-6736(17)32152-9. 28919116 PMC5605883

[3] Liu W , Liu J , Song Y , et al. Mortality of lymphoma and myeloma in China, 2004–2017: an observational study. J Hematol Oncol. 2019;12:22 10.1186/s13045-019-0706-9. 30832702 PMC6399942

[4] Teras LR , DeSantis CE , Cerhan JR , Morton LM , Jemal A , Flowers CR . 2016 US lymphoid malignancy statistics by World Health Organization subtypes. CA Cancer J Clin. 2016;66:443‐459. 10.3322/caac.21357. 27618563

[5] Zeng H , Chen W , Zheng R , et al. Changing cancer survival in China during 2003–15: a pooled analysis of 17 population‐based cancer registries. Lancet Glob Health. 2018;6:e555‐e567. 10.1016/S2214-109X(18)30127-X. 29653628

[6] Yang Y , Cao J‐Z , Lan S‐M , et al. Association of improved locoregional control with prolonged survival in early‐stage extranodal nasal‐type natural killer/T‐cell lymphoma. JAMA Oncol. 2017;3:83‐91. 10.1001/jamaoncol.2016.5094. 27893001

[7] Allemani C , Matsuda T , Di Carlo V , et al. Global surveillance of trends in cancer survival 2000–14 (CONCORD‐3): analysis of individual records for 37 513 025 patients diagnosed with one of 18 cancers from 322 population‐based registries in 71 countries. Lancet. 2018;391:1023‐1075. 10.1016/S0140-6736(17)33326-3. 29395269 PMC5879496

[8] Wang C , Rao K , Wu S , Liu Q . Health care in China: improvement, challenges, and reform. Chest. 2013;143:524‐531. 10.1378/chest.12-1839. 23381317

[9] Meng Q , Xu L , Zhang Y , et al. Trends in access to health services and financial protection in China between 2003 and 2011: a cross‐sectional study. Lancet. 2012;379:805‐814. 10.1016/S0140-6736(12)60278-5. 22386034

[10] Bhatt VR , Dhakal P , Dahal S , et al. Demographic and other characteristics of nodal non‐Hodgkin's lymphoma managed in academic versus non‐academic centers. Ther Adv Hematol. 2015;6:223‐227. 10.1177/2040620715592568. 26425335 PMC4556968

[11] Liu W , Liu J , Song Y , et al. Burden of lymphoma in China, 2006–2016: an analysis of the Global Burden of Disease Study 2016. J Hematol Oncol. 2019;12:115 10.1186/s13045-019-0785-7. 31744509 PMC6862726

[12] Broccoli A , Zinzani PL . The role of transplantation in Hodgkin lymphoma. Br J Haematol. 2018;184:93‐104. 10.1111/bjh.15639. 30407612

[13] Yang M , Ping L , Liu W , et al. Clinical characteristics and prognostic factors of primary extranodal classical Hodgkin lymphoma: a retrospective study. Hematology. 2019;24:413‐419. 10.1080/16078454.2019.1598678. 30922173

[14] Kwan A , Chadwick N , Hancock B . Improving survival of patients with hodgkin lymphoma over 4 decades: experience of the british national lymphoma investigation (BNLI) with 6834 patients. Clin Lymphoma Myeloma Leuk. 2017;17:108‐119. 10.1016/j.clml.2016.11.004. 28027894

[15] Zhang Y , Zhang J , Zeng H , Zhou XH , Zhou HB . Nomograms for predicting the overall and cancer‐specific survival of patients with classical Hodgkin lymphoma: a SEER‐based study. Oncotarget. 2017;8:92978‐92988. 10.18632/oncotarget.21722. 29190971 PMC5696237

[16] Pfreundschuh M , Kuhnt E , Trümper L , et al. CHOP‐like chemotherapy with or without rituximab in young patients with good‐prognosis diffuse large‐B‐cell lymphoma: 6‐year results of an open‐label randomised study of the MabThera International Trial (MInT) Group. Lancet Oncol. 2011;12:1013‐1022. 10.1016/S1470-2045(11)70235-2. 21940214

[17] Wu J‐Q , Song Y‐P , Su L‐P , et al. Three‐year follow‐up on the safety and effectiveness of rituximab plus chemotherapy as first‐line treatment of diffuse large b‐cell lymphoma and follicular lymphoma in real‐world clinical settings in China: A Prospective, Multicenter Noninterventional Study. Chin Med J (Engl). 2018;131:1767‐1775. 10.4103/0366-6999.237401. 30058572 PMC6071449

[18] Soini EJ , Martikainen JA , Nousiainen T . Treatment of follicular non‐Hodgkin's lymphoma with or without rituximab: cost‐effectiveness and value of information based on a 5‐year follow‐up. Ann Oncol. 2011;22:1189‐1197. 10.1093/annonc/mdq582. 21135053 PMC3082160

[19] Auweiler PW , Müller D , Stock S , Gerber A . Cost effectiveness of rituximab for non‐Hodgkin's lymphoma: a systematic review. Pharmacoeconomics. 2012;30:537‐549. 10.2165/11591160-000000000-00000. 22612993

[20] Danese MD , Reyes CM , Gleeson ML , Halperin M , Skettino SL , Mikhael J . Estimating the population benefits and costs of rituximab therapy in the United States from 1998 to 2013 using real‐world data. Med Care. 2016;54:343‐349. 10.1097/MLR.0000000000000486. 26759977 PMC4795096

[21] Yang H , Wu M , Shen YE , et al. Treatment strategies and prognostic factors of primary gastric diffuse large B cell lymphoma: a retrospective multicenter study of 272 cases from the China lymphoma patient registry. Int J Med Sci. 2019;16:1023‐1031. 10.7150/ijms.34175. 31341416 PMC6643119

[22] Horwitz SM , Zelenetz AD , Gordon LI , et al. Guidelines insights: non‐Hodgkin's lymphomas, version 3.2016. J Natl Compr Canc Netw. 2016;14:1067‐1079. 27587620 10.6004/jnccn.2016.0117

[23] d'Amore F , Gaulard P , Trümper L , et al. Peripheral T‐cell lymphomas: ESMO clinical practice guidelines for diagnosis, treatment and follow‐up. Ann Oncol. 2015;26(Suppl 5):v108‐v115. 10.1093/annonc/mdv201. 26314772

[24] Carson KR , Horwitz SM , Pinter‐Brown LC , et al. A prospective cohort study of patients with peripheral T‐cell lymphoma in the United States. Cancer. 2017;123:1174‐1183. 10.1002/cncr.30416. 27911989 PMC5650190

[25] Bellei M , Foss FM , Shustov AR , et al. The outcome of peripheral T‐cell lymphoma patients failing first‐line therapy: a report from the prospective, International T‐Cell Project. Haematologica. 2018;103:1191‐1197. 10.3324/haematol.2017.186577. 29599200 PMC6029527

[26] Liu X , Yang M , Wu M , et al. A retrospective study of the CHOP, CHOPE, and CHOPE/G regimens as the first‐line treatment of peripheral T‐cell lymphomas. Cancer Chemother Pharmacol. 2019;83:443‐449. 10.1007/s00280-018-3744-z 30511217

[27] Wu M , Wang X , Xie Y , et al. Outcome and prospective factor analysis of high‐dose therapy combined with autologous peripheral blood stem cell transplantation in patients with peripheral t‐cell lymphomas. Int J Med Sci. 2018;15:867‐874. 10.7150/ijms.23067. 30008598 PMC6036090

[28] Hester LL , Park SI , Wood WA , Stürmer T , Brookhart MA , Lund JL . Cause‐specific mortality among medicare beneficiaries with newly diagnosed non‐Hodgkin lymphoma subtypes. Cancer. 2019;125:1101‐1112. 10.1002/cncr.31821. 30548238 PMC6719299

[29] Castellino SM , Geiger AM , Mertens AC , et al. Morbidity and mortality in long‐term survivors of Hodgkin lymphoma: a report from the Childhood Cancer Survivor Study. Blood. 2011;117:1806‐1816. 10.1182/blood-2010-04-278796. 21037086 PMC3056636

